# Lithium for Radiation-Induced Damage in Pediatric Brain Tumors: Preclinical Evidence and Clinical Perspectives

**DOI:** 10.3390/jcm15187266

**Published:** 2026-09-18

**Authors:** Francesca Bardi, Antonio Restaino, Claudia Calderoni, Silvia Montanari, Delfina Janiri, Giovanni Camardese, Antonio Maria D’Onofrio, Gabriele Sani, Alessio Simonetti

**Affiliations:** 1 Department of Neuroscience, Section of Psychiatry, Università Cattolica del Sacro Cuore, 00168 Rome, Italy; 2 Department of Neuroscience, Section of Psychiatry, Fondazione Policlinico Universitario Agostino Gemelli IRCCS, 00168 Rome, Italy; 3 Department of Life Science, Health, and Health Professions, Link Campus University, 00165 Rome, Italy; 4 Department of Psychiatry and Behavioral Sciences, Baylor College of Medicine, Houston, TX 77030, USA

**Keywords:** lithium, radiotherapy, pediatric brain tumors, brain radiation, neuroprotection, neurocognition

## Abstract

Central nervous system (CNS) tumors account for nearly one third of pediatric cancers and remain the leading cause of cancer-related mortality in children. Cranial radiotherapy is a cornerstone of treatment for many high-grade and embryonal brain tumors, often in combination with surgery and chemotherapy. However, improved survival has been accompanied by a growing burden of late neurocognitive, emotional, social, and endocrine sequelae. Damage to hippocampal neural stem and progenitor cells partly drives radiation-induced cognitive decline, which leads to reduced neurogenesis and increased gliogenesis. Lithium has emerged as a promising strategy to protect or repair the irradiated brain through its neuroprotective, anti-inflammatory, anti-apoptotic, and pro-neurogenic effects. Preclinical evidence supports its potential to mitigate radiation-induced brain injury. In cellular models, lithium appears to protect neural progenitors and hippocampal neurons from irradiation-related damage but does not confer comparable protection to tumor cells. In juvenile rodent models, lithium administration before, during, or after cranial irradiation has been associated with reduced apoptosis in hippocampal neurogenic regions, preservation or restoration of neurogenesis, attenuation of neuroinflammation, and improvement of cognitive and behavioral outcomes. Importantly, delayed lithium treatment has also shown restorative effects, indicating a potential role as a neuroregenerative strategy for survivors with late effects. Mechanistically, these benefits appear to arise from modulation of apoptotic pathways, GSK-3β-related signaling, neurogenic cell fate, and epigenetic programs that promote neuronal differentiation over gliogenesis. These data have provided the rationale for early clinical translation and ongoing trials now assessing whether lithium can improve neurocognitive outcomes after cranial radiotherapy in pediatric brain tumor patients. This narrative review summarizes the preclinical basis and emerging clinical evidence for lithium as a potential adjunct to radiotherapy while discussing key unresolved issues, including optimal timing, treatment duration, tumor safety, and patient selection.

## 1. Introduction

Tumors of the central nervous system (CNS) are one of the leading causes of death from cancer in children. Better survival over the past decades has been achieved in many pediatric brain tumors by improvements in neurosurgery, chemotherapy, radiotherapy, molecular stratification, and supportive care [1]. However, these gains have also led to an increasing burden of treatment-related morbidity in long-term survivors [2]. Children treated for brain tumors may have lasting neurocognitive, psychological, and endocrine sequelae that can significantly affect education, employment, and quality of life [3,4].

Cranial radiotherapy remains a cornerstone of treatment for several pediatric CNS malignancies, including medulloblastoma and other embryonal tumors, ependymoma, high-grade glioma, germ cell tumors, and selected unresectable or recurrent low-grade gliomas. Although contemporary radiation approaches aim to reduce exposure of healthy brain tissue, neurocognitive impairment remains a major clinical concern and results from the exposure of developmental tissue [5]. Long-term deficits may involve global intellectual functioning, processing speed, attention, working memory, executive functions, learning, and episodic memory, often worsening over time [5]. Younger age at treatment, higher radiation dose, larger irradiated volumes, hydrocephalus, tumor location, and concomitant chemotherapy have been linked to increased vulnerability to late neurocognitive effects.

The biological mechanisms underlying radiation-induced cognitive decline are multifactorial. Cranial irradiation induces direct DNA damage, oxidative stress, apoptosis, endothelial dysfunction, white matter injury, neuroinflammation, altered synaptic plasticity, and endocrine disturbances [5,6,7]. A particularly relevant target is the neurogenic niche of the hippocampus. Neural stem and progenitor cells located in the subgranular zone of the dentate gyrus, together with progenitor populations in the subventricular zone, have high proliferative activity and are therefore especially vulnerable to irradiation [8]. Damage to these populations may result in reduced neurogenesis and impaired neuronal differentiation, with changes in the hippocampal microenvironment. In parallel, irradiation activates microglia and astrocytes, sustaining inflammatory signaling and further compromising neural progenitors and neural circuits [9]. These processes provide a biologically plausible link between cranial irradiation and the persistent impairments in learning, memory, and executive functions observed in pediatric brain tumor survivors [5].

Existing approaches to reduce radiation-induced neurotoxicity involve technical improvements in radiotherapy planning and dose delivery and attempts to reduce treatment intensity for lower-risk disease. In addition to their importance, these approaches cannot completely prevent injury to normally developing brain tissue [10]. Therefore, there is a significant unmet need for pharmacological interventions that can prevent, attenuate, or reverse radiation-induced damage without compromising tumor control.

Lithium is a particularly promising candidate in this context. Widely used in psychiatry, lithium has a long-established clinical pharmacology and a well-characterized safety profile when appropriately monitored [11]. In addition to psychiatric indications, lithium has shown neuroprotective, anti-apoptotic, anti-inflammatory, and pro-neurogenic effects in several experimental models of neurological injury [12,13]. The wide range of lithium’s biological mechanisms has not yet been fully elucidated, but its pleiotropic actions are thought to include modulation of several intracellular pathways, such as inhibition of glycogen synthase kinase-3β (GSK-3β), activation of pro-survival signaling cascades, regulation of neurotrophic factors, and effects on neurogenesis, synaptic plasticity, mitochondrial function, and inflammatory processes [14]. These mechanisms become interestingly relevant to the cellular consequences of cranial irradiation. Although lithium has previously been proposed as a potential neuroprotective agent against radiation-induced brain injury, evidence is still preliminary, heterogeneous, and scattered across cellular models, animal studies, and a small body of clinical and translational research. Moreover, no recent review has synthesized these findings and addressed the specific challenges of translating lithium from preclinical models to clinical applications in the context of cranial radiotherapy for pediatric brain tumors.

This narrative review summarizes the available evidence on lithium as a potential neuroprotective and neuroregenerative adjunct to cranial radiotherapy in pediatric brain tumors. The first section examines in vitro studies that investigate the cellular and molecular effects of lithium after irradiation. The second section focuses on in vivo models of cranial irradiation with emphasis on neurogenesis, inflammation, apoptosis, and functional cognitive consequences. Finally, the review explores the limited existing clinical evidence and ongoing translational studies, highlighting the key challenges that need to be overcome before lithium can be incorporated into routine neuro-oncological practice.

### Review Approach

This narrative review provides a qualitative synthesis of preclinical and clinical evidence on lithium in the context of radiation-induced brain injury, with particular attention to its relevance to pediatric brain tumors. The evidence is discussed across cellular models, animal studies, and clinical investigations, focusing on treatment timing, neuroprotective and neuroregenerative effects, and oncological safety. A reproducible search and screening record is unavailable.

## 2. Evidence of Lithium-Mediated Radioprotection

### 2.1. In Vitro Evidence

In vitro studies provide the first mechanistic rationale for considering lithium as a selective neuroprotective strategy in the context of cranial irradiation (Table 1). Data are available from murine hippocampal neuronal cells, rodent neural stem and progenitor cells (NSPCs), human induced pluripotent stem cell (iPSC)-derived NSPCs, and pediatric brain tumor cell lines, including medulloblastoma, primitive neuroectodermal tumor, and glioma models [15,16,17,18,19]. These studies suggest that lithium may differentially modulate the radiation response of normal neural cells and malignant cells, despite differences in their cellular origin, developmental stage, irradiation dose, lithium concentration, treatment timing, and readout measures.

In models of neuronal and progenitor cells, lithium exposure prior to irradiation has been shown to increase cell survival and decrease apoptosis [15,16]. In these studies, hippocampal neuronal cells were pretreated with lithium chloride (LiCl) at 3 mM for 7 days before irradiation [15,16]. In hippocampal neuronal cells, these effects were associated with activation of Akt-dependent pro-survival signaling, GSK-3β inhibition, and a shift to a more anti-apoptotic molecular profile, including an increased Bcl-2/Bax ratio [15]. Pre-treatment with lithium has also been associated with improved repair of irradiation-induced DNA double-strand breaks via DNA-PK-dependent non-homologous end joining, with less persistence of DNA damage markers after irradiation in hippocampal neurons [16]. These results suggest that lithium may mitigate the short-term cellular effects of irradiation via the activation of pro-survival pathways, suppression of apoptosis, and more efficient repair of DNA double-strand breaks.

On the other hand, lithium also mediates radioprotective effects beyond acute phases. Studies in irradiated NSPCs have shown that lithium can affect cell cycle regulation, proliferative capacity, and fate commitment after irradiation. More specifically, lithium has been linked to a partial reversal of irradiation-induced cell cycle arrest, an increased percentage of cells entering S phase, enhanced growth of neurospheres, and reduced residual DNA damage in irradiated hippocampal NSPCs, treated with 3 mM LiCl beginning 12 h before irradiation, with gene expression assessed 24 h after irradiation [17]. These effects are particularly important in the context that cranial irradiation may affect neurocognitive development not only through immediate cell loss but also through sustained depletion of neural progenitor pools and reduced capacity for hippocampal neurogenesis. More recent transcriptomic and epigenetic studies have expanded current knowledge, suggesting that lithium may actively reprogram the differentiation program of irradiated progenitors. In rodent NSPCs treated with 3 mM LiCl beginning 12 h before irradiation, with gene expression assessed 24 h after irradiation, lithium exposure modulated genes related to cell cycle regulation and neuronal signaling, including Tppp and Gad2/Gad65, along with changes in DNA methylation patterns [20]. Consistent with these findings, in human iPSC-derived NSPCs exposed to 3 mM LiCl for 3 h, starting 1 h after irradiation, lithium was associated with widespread alterations in gene expression and DNA methylation, including increased expression of pro-neuronal genes such as GAD2 and modulation of genes related to BMP signaling and gliogenic differentiation, such as GREM1, SOX5, SOX10, and PTN [19]. These findings are consistent with a model where lithium may promote a more neurogenic and less gliogenic response to irradiation, favoring long-term restoration of hippocampal circuitry rather than simply promoting short-term cell viability.

This suggests that the timing of lithium administration is likely to be biologically relevant. The main purpose of pretreatment paradigms is to inform about acute radioprotection, whereas the post-irradiation paradigms are used to answer the question of whether lithium can improve repair and regenerative responses after damage has already occurred. This latter approach may be especially attractive from a translational perspective, as delayed administration could theoretically alleviate concerns about lithium interfering with tumor-directed radiation effects while still allowing for the potential to promote neural recovery [19].

One important question is whether lithium may also protect tumor cells from radiation. Available in vitro data do not indicate a generalized tumor radioprotective effect. However, in cell lines originating from gliomas and medulloblastomas, lithium did not improve clonogenic survival or maintain viability after irradiation, even though neural progenitor populations showed relatively higher resistance to lithium-induced toxicity [15,18]. In the combined lithium-irradiation experiments by Zhou et al., cells received 1 or 10 mM LiCl approximately 24 h before irradiation, and viability was assessed 72 h after irradiation [18]. Lithium was associated with reduced tumor-cell viability in several pediatric brain tumor models. Indeed, in a particular molecular context, radiosensitization was investigated using 2 mM LiCl administered for 24 h before irradiation, and lithium-mediated activation of WNT was shown to abrogate the radioresistance associated with TP53 mutation in medulloblastoma, suggesting a possible radiosensitizing rather than tumor-protecting effect [21]. However, these findings should not be taken as evidence that lithium has antitumoral properties in pediatric brain tumors. Tumor subtype, molecular profile, basal WNT activity, TP53 status, lithium concentration, and also the schedule of administration may all influence the direction and magnitude of the observed effects.

Collectively, in vitro studies suggest the existence of a potential therapeutic window where lithium protects irradiated neural cells and progenitors without impairing and, in some instances, potentially enhancing the cytotoxic effects of radiotherapy on tumor cells. Their translational relevance depends on validation in tumor-bearing animal models capable of simultaneously evaluating neuroprotection and tumor control.

**Table 1:** In vitro studies.

Study	Cellular Model	Irradiation and Lithium Paradigm	Outcomes in Neural Cells	Outcomes in Tumor Cells	Proposed Mechanisms
Yazlovitskaya et al., 2006 [15]	Murine hippocampal HT-22 cells; murine GL261 and human D54 glioma cells	LiCl 3 mM for 7 days before irradiation; 0–8 Gy clonogenic assays	Increased survival and reduced apoptosis of irradiated HT-22 cells	No increase in clonogenic survival of GL261 or D54 cells	Akt activation, GSK-3β inhibition, increased Bcl-2/Bax ratio; early evidence of differential radioprotection
Yang et al., 2009 [16]	Hippocampal neural cells and glioma models	LiCl 3 mM for 7 days before 3 Gy irradiation	Reduced DNA damage and apoptosis in hippocampal cells	No comparable protection in glioma cells	Enhanced DNA-PK-dependent non-homologous end joining; improved repair of radiation-induced DNA double-strand breaks
Zhukova et al., 2014 [21]	Medulloblastoma models, including TP53-mutant cells	2 mM LiCl administered for 24 h before irradiation	Not the primary focus	Reduced radioresistance in TP53-mutant medulloblastoma models	Suggests context-dependent radiosensitization rather than tumor radioprotection
Zanni et al., 2015 [17]	Rodent hippocampal NSPCs	LiCl 1 or 3 mM 12 h before 3.5 Gy irradiation; assessments at 6–72 h after irradiation	Increased proliferation, partial reversal of cell cycle arrest, increased neurosphere growth	NA	Suggests restoration of regenerative capacity rather than only anti-apoptotic protection
Zhou et al., 2017 [18]	Human medulloblastoma cell lines, PNET cells, murine C17.2 neural progenitors	LiCl 1 or 10 mM for 24 h before 5 or 10 Gy irradiation; viability assessed 72 h later	Neural progenitor cells relatively resistant to lithium toxicity	Lithium did not protect irradiated medulloblastoma cells; reduced viability in several tumor lines	Supports a potential therapeutic window between neural progenitors and pediatric tumor cells
Neofytou et al., 2023 [19]	Human iPSC-derived NSPCs	LiCl 3 mM for 3 h, starting 1 h after 4 Gy irradiation; subsequent expansion for 48 h in standard medium	Altered gene expression and DNA methylation profiles consistent with a shift toward neurogenesis and away from gliogenesis	NA	Epigenetic and transcriptional modulation of neuronal and glial differentiation programs, including GAD2 and BMP-related genes
Zanni et al., 2021 [20]	Rodent embryonic telencephalic NSPCs	LiCl 3 mM 12 h before irradiation; 2.5 Gy irradiation	Modulation of cell cycle and neuronal signaling genes; methylation changes involving *Tppp* and *Gad2/Gad65*	NA	Indicates epigenetic regulation of progenitor-cell fate after irradiation

*Abbreviations*. Akt, protein kinase B; Bax, B-cell lymphoma 2-associated X protein; Bcl-2, B-cell lymphoma 2; BMP, bone morphogenetic protein; DNA, deoxyribonucleic acid; DNA-PK, DNA-dependent protein kinase; GAD2/Gad2, glutamate decarboxylase 2; GAD65, 65-kDa glutamate decarboxylase, encoded by GAD2/Gad2; GSK-3β, glycogen synthase kinase-3 beta; Gy, gray; h, hour; iPSC, induced pluripotent stem cell; kDa, kilodalton; LiCl, lithium chloride; mM, millimolar; NA, not assessed; NSPCs, neural stem and progenitor cells; PNET, primitive neuroectodermal tumor; TP53, tumor protein p53; Tppp, tubulin polymerization-promoting protein. HT-22, GL261, D54, and C17.2 are cell line identifiers.

### 2.2. In Vivo Evidence

In vivo studies suggest that lithium may modulate several interconnected components of radiation-induced brain injury, rather than simply acting as an anti-apoptotic agent. In neonatal and juvenile rodent models, lithium has been associated with decreased cell death in vulnerable hippocampal populations, increased repair of DNA damage induced by radiation, preservation of pools of neural progenitor cells, modulation of glial and inflammatory responses, and improved select behavioral and cognitive outcomes. The models are variable in terms of species, developmental stage, radiation dose, lithium regimen, and length of follow-up, but together, they suggest that lithium may have a dual role in both limiting the initial injury and supporting subsequent neural recovery (Table 2).

In the initial stage of radiation-induced damage, proliferating cells within the subgranular zone (SGZ) of the hippocampus show significant susceptibility. In neonatal and juvenile rodents, lithium pretreatment attenuated irradiation-induced apoptosis in this neurogenic niche and correlated with a subsequent improvement in hippocampal-dependent cognitive performance. In the study by Yazlovitskaya et al., administration of lithium prior to cranial irradiation reduced apoptosis in the subgranular zone and improved performance in the Morris water maze several weeks after treatment, providing the first in vivo evidence that preservation of hippocampal cells may result in functional benefit [15]. Later mechanistic work showed that lithium increased the repair of radiation-induced DNA double-strand breaks in hippocampal neurons via a DNA-PK-dependent non-homologous end-joining pathway, supporting this protective effect. In irradiated mice, pretreatment with lithium was associated with reduced persistence of DNA damage, but its anti-apoptotic effect was markedly reduced when DNA-PK activity was genetically abolished or pharmacologically inhibited [16]. Further evidence of this protective effect on the developing hippocampus was provided by Huo et al., who studied lithium in an immature mouse model of cranial irradiation. Lithium decreased apoptosis of neural progenitor cells in the dentate gyrus and was associated with a less pronounced impairment of the growth of the granule cell layer. In addition, lithium-treated animals showed less microglial activation and better functional performance following irradiation, supporting the concept that preservation of the neurogenic niche could impact both tissue and behavioral effects of radiation exposure [22]. However, the reduced microglial activation could be a direct anti-inflammatory effect of lithium or a consequence of the reduced cell death and tissue damage at early time points. Consistent with the in vitro evidence, data from in vivo studies of lithium pretreatment suggest that lithium-mediated neuroprotection involves interconnected mechanisms, including enhanced repair of radiation-induced DNA lesions and modulation of apoptotic and pro-survival signaling.

In addition to acute radioprotection, lithium may contribute to the preservation of cellular processes involved in longer-term hippocampal recovery. In juvenile rats, lithium reduced irradiation-induced progenitor-cell death in the subgranular zone during the first 24 h after irradiation, and at 16 weeks, lithium-treated animals showed greater survival of newly generated cells and attenuated reductions in both neurogenesis and astrogenesis. These effects were accompanied by improvements in irradiation-induced memory deficits and motor hyperactivity and partial protection against hypothalamic-pituitary function disorders [18]. A subsequent study in juvenile mice reported that lithium enhanced progenitor cell proliferation and differentiation following cranial irradiation and attenuated irradiation-associated loss of oligodendrocytes in the dentate gyrus [23]. However, the addition of a synthetic retinoid receptor agonist did not significantly augment lithium-associated recovery, suggesting the interaction of lithium, inflammation, and regenerative signaling is likely more complex than a mere additive anti-inflammatory effect. Taken together, these findings suggest that lithium may act beyond the acute prevention of progenitor-cell death. Its longer-term effects may involve preservation of the capacity of neural progenitors to contribute to neuronal and glial replenishment, protection of oligodendroglial populations, and partial restoration of a cellular environment more supportive of hippocampal maturation and plasticity. This broader action may be particularly relevant because radiation-induced cognitive impairment is unlikely to result solely from reduced neurogenesis but may also reflect impaired glial support and persistent alterations in the local microenvironment.

Timing of lithium treatment appears to be particularly important for translational development. Most early studies used pretreatment paradigms that are useful for radioprotection but are of concern if there is the theoretical possibility of protecting tumor cells during radiotherapy. Delayed-treatment studies address this limitation by examining whether lithium can aid in recovery after radiation damage has already occurred. In a juvenile mouse model, lithium administered several weeks after whole-brain irradiation increased neural progenitor proliferation and improved the maturation-associated morphology of immature hippocampal neurons. In the same study, neuronal differentiation and integration became evident only after lithium treatment was discontinued. This finding suggests that lithium exposure may initially expand the progenitor-cell population, whereas its subsequent withdrawal may permit these cells to progress toward neuronal maturation and integration. In addition, delayed lithium treatment was associated with improved spatial learning and memory retention, suggesting a neuroregenerative role that may extend beyond acute protection from apoptosis [20].

In summary, the in vivo literature supports a model where lithium may reduce early radiation-induced DNA damage and apoptosis, maintain neural progenitor-cell populations, promote neuronal and glial recovery, and improve selected cognitive and neuroendocrine outcomes. However, there is still limited evidence due to the prevalence of non-tumor-bearing juvenile rodent models, heterogeneous lithium schedules, and the frequent use of single-dose irradiation paradigms that do not fully replicate modern fractionated radiotherapy. Moreover, it is still difficult to discriminate the relative contribution of single mechanisms, such as DNA repair, inflammation, progenitor cell proliferation, and circuit integration. Therefore, future preclinical studies should investigate the use of lithium in tumor-bearing models with clinically relevant radiation schedules and simultaneously evaluate neuroprotection, neurocognition, and tumor control.

### 2.3. Human Studies

Despite the extensive preclinical literature supporting lithium as a neuroprotective strategy against radiation-induced brain injury, clinical evidence remains remarkably limited (Table 3). A narrative review published by Khasraw et al. in 2012 [24] highlighted the marked gap between promising experimental findings and their clinical translation, identifying only one prospective human study available at that time. To date, only a small number of studies have evaluated lithium in human subjects within the context of cranial irradiation.

The first prospective clinical experience was reported in a phase I feasibility study presented at the ASTRO Annual Meeting in 2007 [25]. Five adult patients with brain metastases from extracranial malignancies received lithium carbonate starting one week before and continuing throughout whole-brain radiotherapy (30 Gy in 10 fractions). Treatment compliance was 100%, the median serum lithium concentration reached 0.6 mEq/L, and no grade 3 or higher non-hematological toxicities were observed. Although the study was not designed to assess efficacy, serial neurocognitive assessments reportedly showed no decline in short-term memory during treatment. The authors concluded that concurrent lithium administration during cranial irradiation was feasible and well tolerated, supporting further clinical investigation.

Subsequently, a phase I/II clinical trial evaluated lithium as a potential neuroprotective strategy in patients with small-cell lung cancer undergoing prophylactic cranial irradiation (PCI) (NCT01553916) [26]. Patients received lithium carbonate 300 mg orally twice daily, starting 7 days before the initiation of PCI and continuing throughout the course of irradiation, with the primary aim of assessing the feasibility and safety of this approach. Secondary objectives included the evaluation of neurocognitive performance, particularly verbal memory, using standardized neuropsychological assessments such as the Hopkins Verbal Learning Test. The study demonstrated that lithium administration was feasible and generally well tolerated in this setting. However, the small sample size, single-arm design, and absence of a control group precluded robust conclusions regarding its neuroprotective efficacy [26]. Although the trial provided important proof of clinical feasibility and represented an important step toward clinical translation of the preclinical findings, detailed neurocognitive outcome data remain limited, and no subsequent randomized confirmatory trials have been conducted.

More recently, clinical development has expanded to pediatric neuro-oncology. The ongoing LiBRA trial (NCT06051240) is a randomized, double-blind, placebo-controlled phase II study evaluating whether six months of lithium treatment can prevent or attenuate late neurocognitive impairment in pediatric brain tumor survivors previously treated with cranial radiotherapy [27]. Specifically, oral lithium sulfate (Lithionit) is initiated at 42 mg elemental lithium (6 mmol) once daily and gradually titrated to serum concentrations of 0.5–1.0 mmol/L; treatment starts after radiotherapy, with no fixed interval. The trial is designed to assess cognitive performance, quality of life, neuroimaging outcomes, and treatment safety, representing the first dedicated randomized study specifically targeting radiation-induced cognitive sequelae in this population. At present, efficacy results have not yet been published.

Overall, despite nearly two decades of promising experimental evidence, clinical translation remains at an early stage. Current human data are largely restricted to early-phase safety studies and ongoing clinical trials, and no adequately powered randomized study has yet demonstrated that lithium improves neurocognitive outcomes following cranial irradiation. Therefore, the results of ongoing trials will be crucial in determining whether the robust neuroprotective effects observed in experimental models can be translated into meaningful clinical benefits for pediatric brain tumor survivors.

## 3. Discussion

Available evidence supports lithium as a biologically plausible candidate for mitigating radiation-induced injury in the brain during developmental phases, but its potential value may reside less in a single dominant mechanism than in its ability to modulate several temporally distinct components of radiation damage. The biologically heterogeneous mechanisms of lithium’s action are especially pertinent to cranial irradiation, since late cognitive impairment is unlikely to stem from a singular lesion or cell type but rather appears to be the consequence of cumulative impacts [5,6,7,18,22,23].

An important implication of the studies explored in this review is that the timing of lithium administration may determine both the biological effects achieved and the associated oncological risks. Pretreatment primarily targets acute radiation-induced injury, including apoptosis and DNA double-strand breaks, and may therefore be most effective when administered before or during irradiation [15,16,22]. This approach still leaves the concern that a drug that can spare normal neural tissue may also reduce tumor responses to radiotherapy. The available in vitro evidence does not indicate a generalized radioprotective effect of lithium in glioma or medulloblastoma cells. In selected molecular contexts, lithium may even enhance tumor-cell radiosensitivity [15,18,21]. The effect of lithium on tumor control is likely dependent on tumor histology, molecular subgroup, baseline WNT/GSK-3β signaling, TP53 status, timing of radiation, and drug exposure [18,21]. The lack of evidence for radioprotection of the tumor cannot be considered definitive proof of oncological safety for now. By contrast, delayed lithium treatment may provide a conceptually different and potentially more translatable strategy. The postirradiation studies reviewed here suggest that lithium can promote subsequent neuronal maturation, stimulate progenitor proliferation, and improve cognitive performance even when treatment occurs after acute radiation injury [17,19,20]. This raises the possibility that lithium may be used to prevent injury and modify established neurodevelopmental dysfunction. Importantly, this approach may mitigate concerns of interference with tumor-directed radiation effects, especially in patients who have completed radiotherapy and are clinically stable. The possibility that neuronal differentiation and circuit integration might be more evident after lithium discontinuation also suggests that continuous exposure might not be the optimal therapeutic regimen [20]. However, this rationale derives from preclinical findings, particularly the neuronal maturation observed after lithium withdrawal in the mouse component of the study by Zanni et al. [20]. No clinical neuroprotection regimen involving repeated, planned lithium interruptions to promote neuronal maturation was identified in the oncology literature reviewed. Future studies should examine whether time-limited or intermittent administration can achieve a better balance between progenitor proliferation, neuronal differentiation, and long-term network integration.

Another important point to consider is that the clinical target should not be defined only as hippocampal neurogenesis. Much of the preclinical rationale for lithium relies on hippocampal progenitor-cell models, but radiation-induced cognitive impairment in pediatric brain tumor survivors is more than just memory dysfunction. Partially distinct mechanisms may mediate changes in processing speed, attention, executive functioning, working memory, endocrine function, and psychosocial adaptation [28,29,30]. In addition, some neurocognitive vulnerabilities may be present before radiotherapy due to the tumor itself or its consequences, including treatments [30,31,32]. This finding has important implications for trial design: treatment effects should be assessed against longitudinal developmental trajectories and baseline neurocognitive status, not just against cross-sectional post-treatment assessments [29]. There are good clinical reasons for processing speed to have been chosen as the primary endpoint in LiBRA, but in future trials it would also be important to determine whether any lithium-related benefit is domain-specific, generalized, or limited to patients with particular patterns of radiation dose distribution and developmental risk profiles [27,33].

The future clinical role of lithium is also likely to be tied to its incorporation rather than supersession of modern radiotherapy optimization. Advances in conformal planning, proton therapy, and dose reduction strategies may reduce exposure to vulnerable brain structures but cannot eliminate injury to children requiring cranial or craniospinal irradiation [34,35,36]. Therefore, pharmacologic neuroprotection may be most useful for those at greatest residual risk after modern planning techniques, including younger children, those receiving higher doses or larger irradiated volumes, and patients with substantial exposure of the hippocampus, temporal lobes, white matter, or hypothalamic-pituitary axis [5,29,34]. This points to a future framework of precision neuroprotection, where lithium would be evaluated in the context of individual dosimetry, molecular tumor features, baseline neurocognitive profile, neuroimaging markers, and endocrine risk. Lithium’s potential clinical role should also be considered alongside other pharmacological approaches to neuroprotection, particularly memantine. In adults receiving whole-brain radiotherapy, the randomized RTOG 0614 trial showed that memantine delayed cognitive decline, although the primary endpoint of delayed recall at 24 weeks did not reach statistical significance [37]. More recently, a randomized pediatric pilot trial provided preliminary findings supporting further investigation in younger patients [38]. These studies provide a clinical context for evaluating lithium, but the potential advantage of lithium over memantine has yet to be demonstrated.

Safety is another important translational issue. Lithium is well known in clinical practice, is taken by mouth, and is familiar to pediatric and psychiatric practice. At the same time, due to its narrow therapeutic index, systematic monitoring of serum concentrations, renal function, thyroid function, hydration status, and potential drug interactions is required [39,40,41]. These concerns may be particularly relevant in children exposed to corticosteroids, nephrotoxic chemotherapy, vomiting, poor oral intake, electrolyte disturbances, or endocrine dysfunction secondary to tumor treatment [42,43]. In this context, concomitant corticosteroid treatment warrants specific consideration given their wide use in oncological settings. In cultured adult rat hippocampal neural precursor cells, lithium counteracted dexamethasone-induced suppression of proliferation, but this preclinical finding does not establish a neuroprotective benefit of the combination in patients undergoing radiotherapy [44]. Concomitant antiseizure therapy also warrants caution. Clinical experience outside neuro-oncology suggests that lithium can be used in selected patients with epilepsy [45,46]; however, neurotoxicity has been reported with certain antiseizure drug combinations, notably carbamazepine, even at therapeutic plasma concentrations [47,48]. Careful medication review and monitoring for neurological toxicity are therefore essential, as serum lithium concentrations alone may not exclude adverse effects. Thus, future trials should demonstrate not only cognitive benefit but also establish the minimum effective exposure, optimal duration of treatment, discontinuation strategies, and patient groups in which monitoring burden or toxicity may outweigh potential benefit [49].

Beyond systemic tolerability, oncological safety remains a key unresolved issue. Most of the preclinical irradiation studies reviewed here used healthy, non-tumor-bearing juvenile rodents exposed to a single radiation dose, limiting their ability to establish oncological safety [15,16,18,20,22,23]. Tumor-induced changes in the surrounding brain, including mechanical compression, impaired vascular perfusion, and neuronal injury, introduce additional factors absent from healthy-brain models [50]. Furthermore, experimental comparisons of fractionated and single-dose irradiation indicate that the exposure schedule influences hippocampal neurogenesis and glial inflammatory responses [51]. These findings raise the possibility that lithium may have different effects under these conditions. Before widespread clinical adoption, lithium therefore requires evaluation in tumor-bearing animal models receiving clinically relevant fractionated radiotherapy, with concurrent assessment of neurogenesis, cognitive outcomes, tumor growth, recurrence, and survival.

At this time, lithium should be considered an experimental approach to radiation-associated neurocognitive injury rather than a therapy ready for routine clinical use. Nevertheless, the preclinical literature provides an unusually consistent signal across apoptosis, DNA repair, progenitor cell biology, neurogenesis, and glial recovery and behavior [15,16,18,20,22,23]. The question is now not whether lithium can impact radiation-induced damage in experimental systems but whether these effects can be translated into clinically meaningful preservation of developmental and cognitive trajectories without compromising oncological outcomes. Answering this question will require the use of tumor-bearing and fractionated-radiotherapy animal models, biomarker-informed clinical trials, standardized neurocognitive assessments, long-term follow-up, and concurrent assessment of tumor control and systemic safety.

## 4. Conclusions

Lithium has the potential to prevent and treat radiation-induced neurocognitive injury in children who survive brain tumors. Experimental evidence suggests lithium may reduce acute DNA damage and apoptosis and support later recovery of progenitor cells, maturation of neurons and glia, and selected cognitive outcomes [15,16,18,20,22]. The distinction between acute radioprotection and delayed neuroregeneration is particularly relevant because postirradiation treatment options could provide a better balance between neural recovery and oncological safety [19,20]. However, current evidence is mainly preclinical and is limited by the heterogeneity of animal models, the single-dose irradiation paradigms, and the absence of adequate tumor-bearing studies. Human data are limited to small early-phase experience and an ongoing randomized pediatric trial [24,27]. The LiBRA trial (NCT06051240) provides a concrete opportunity to evaluate whether post-radiotherapy lithium treatment improves processing speed and broader neurocognitive outcomes in pediatric brain tumor survivors [27]. Building on this approach, future randomized, placebo-controlled trials should incorporate baseline and longitudinal assessments of processing speed, attention, executive functioning, and memory, with prespecified analyses according to age at irradiation and radiation dose distribution. Long-term follow-up should assess the persistence of any cognitive benefit alongside tumor recurrence and systemic safety. Until these clinical questions are resolved, lithium should be regarded as a promising, but unproven, element of a broader precision neuroprotection strategy for children undergoing cranial radiotherapy.

## Figures and Tables

**Table 2:** In vivo studies.

Study	Animal Model	Irradiation Paradigm	Lithium Regimen and Timing	Biological Outcomes	Functional Outcomes
Yazlovitskaya et al., 2006 [15]	Juvenile C57BL/6J mouse; confirmatory Sprague-Dawley rat histology	Single-dose cranial irradiation; 7 Gy for cognitive testing, with 1–10 Gy used in histological dose-response experiments	LiCl 40 mg/kg/day i.p. before irradiation	Reduced hippocampal SGZ apoptosis; activation of Akt/GSK-3β signaling; increased Bcl-2; reduced Bax	Improved hidden-platform Morris water maze performance 6 weeks after irradiation
Yang et al., 2009 [16]	Juvenile male C57BL/6J mouse	Single 3 Gy cranial irradiation after lithium pretreatment	LiCl 40 mg/kg/day i.p. before irradiation	Enhanced DNA-PK-dependent DNA repair; reduced neuronal DNA damage and apoptosis	NA
Huo et al., 2012 [22]	Juvenile male C57BL/6 mouse	Single 8 Gy irradiation of the left cerebral hemisphere on postnatal day 11	LiCl 2 mmol/kg i.p. immediately after irradiation, followed by 1 mmol/kg daily for 6 days	Reduced hippocampal NSPC apoptosis; preservation of neurogenesis; reduced microglial activation	Improved hippocampal-dependent learning, including contextual fear conditioning and IntelliCage place learning
Malaterre et al., 2012 [23]	Juvenile male C57BL/6 mouse	Cranial irradiation in juvenile mice; single-dose paradigm	0.5% Li_2_CO_3_ chow for 1 or 4 weeks initiated after irradiation	Increased NSPC proliferation and regenerative response despite inflammation	NA
Zhou et al., 2017 [18]	Juvenile male Wistar rat	Single 6 Gy cranial irradiation on postnatal day 11	LiCl administered beginning before irradiation and continued after exposure	Reduced hippocampal progenitor loss; preserved neurogenesis; reduced late pituitary injury	Improved memory, locomotor activity, and anxiety-like behavior
Zanni et al., 2021 [20]	Adult female C57BL/6 mouse	Single 4 Gy whole-brain irradiation on postnatal day 21	0.24% Li_2_CO_3_ chow started 4 weeks after irradiation and continued for 4 weeks	Increased NSPC proliferation; enhanced later neuronal maturation/integration	Improved spatial learning and memory

*Abbreviations.* Akt, protein kinase B; Bax, B-cell lymphoma 2-associated X protein; Bcl-2, B-cell lymphoma 2; DNA, deoxyribonucleic acid; DNA-PK, DNA-dependent protein kinase; GSK-3β, glycogen synthase kinase-3 beta; Gy, gray; i.p., intraperitoneal; LiCl, lithium chloride; Li_2_CO_3_, lithium carbonate; mg/kg/day, milligrams per kilogram of body weight per day; NA, not assessed; NSPC, neural stem and progenitor cell; SGZ, subgranular zone. C57BL/6 and C57BL/6J designate mouse strains; Sprague Dawley and Wistar designate rat strains. IntelliCage is an automated behavioral testing system.

**Table 3:** Human studies.

Trial	Population	Design	Main Findings	Main Limitation
ASTRO 2007 (Phase I) [25]	Five adults with brain metastases	Lithium started before and continued during whole-brain radiotherapy	Feasible and well tolerated; no major toxicity reported	Very small, uncontrolled, abstract-only study
NCT01553916 [26]	Adults with small-cell lung cancer receiving prophylactic cranial irradiation	Phase I/II study of lithium during cranial irradiation	Completed; 19 participants enrolled	No posted or peer-reviewed efficacy results
LiBRA trial (NCT06051240) [27]	Pediatric brain tumor survivors after cranial radiotherapy	Randomized, double-blind, placebo-controlled phase II trial; lithium for 6 months; initially 42 mg elemental lithium (6 mmol) once daily, titrated to serum concentrations of 0.5–1.0 mmol/L	Ongoing; evaluates processing speed, broader neurocognitive outcomes, quality of life, neuroimaging, and safety	Efficacy results not yet available

*Abbreviations.* ASTRO, American Society for Radiation Oncology; LiBRA, Lithium Treatment to Prevent Cognitive Impairment After Brain Radiotherapy; NCT, National Clinical Trial identifier assigned by ClinicalTrials.gov.

## Data Availability

The original contributions presented in this study are included in the article. Further inquiries can be directed to the corresponding author.
